# Chemical composition, antioxidant and hepatoprotective activities of methanol extracts from leaves of *Terminalia bellirica* and *Terminalia sericea* (Combretaceae)

**DOI:** 10.7717/peerj.6322

**Published:** 2019-02-27

**Authors:** Mansour Sobeh, Mona F. Mahmoud, Rehab A. Hasan, Mohamed A.O. Abdelfattah, Samir Osman, Harun-or Rashid, Assem M. El-Shazly, Michael Wink

**Affiliations:** 1 Institute of Pharmacy and Molecular Biotechnology, Heidelberg University, Heidelberg, Germany; 2 Department of Pharmacology and Toxicology, Faculty of Pharmacy, Zagazig University, Zagazig, Egypt; 3 Department of Histology, Faculty of Medicine for Girls, Al Azhar University, Cairo, Egypt; 4 Department of Science, College of Engineering and Technology, American University of the Middle East, Kuwait; 5 Department of Pharmacognosy, Faculty of Pharmacy, October 6 University, Cairo, Egypt; 6 Biotechnology Division, Bangladesh Institute of Nuclear Agriculture Bangladesh, Mymensingh, Bangladesh; 7 Department of Pharmacognosy, Faculty of Pharmacy, Zagazig University, Zagazig, Egypt

**Keywords:** *Terminalia sericea*, *Terminalia bellirica*, HPLC-MS/MS, Anti-apoptotic, Hepatoprotection, Polyphenols

## Abstract

**Background:**

Plants belonging to the genus *Terminalia* such as *Terminalia bellirica* and *Terminalia sericea* are used traditionally to treat several diseases and health disorders. Up to this date, the roots of *Terminalia sericea* and the fruits of *Terminalia bellirica* are the mostly studied plant parts. The phytochemical composition and the biological activities of the leaves of both species are not well identified so far.

**Methods:**

The secondary metabolites of *Terminalia bellirica* and *Terminalia sericea* leaves were identified using HPLC-PDA-MS/MS. The antioxidant activities of the leaves extracts were determined by DPPH and FRAP assays. The hepatoprotective potential was evaluated in rats with D-galactosamine induced liver damage. The effect of the extracts on the expression of the anti-apoptotic marker Bcl-2 was measured in an immunohistochemical study. The most abundant compounds identified in the studied extracts were docked into Bcl-2: Bim (BH3) interaction surface using molecular operating environment software.

**Results:**

A total of 85 secondary metabolites were identified in the leaf extracts of both species. Ellagitannins such as corilagin, chebulagic acid, galloylpunicalagin, and digalloyl-hexahydroxydiphenoyl-hexoside were found to be the major components in *Terminalia bellirica* whereas flavonoid glycosides including quercetin rutinoside and quercetin galloyl-glucoside were highly abundant in *Terminalia sericea*. The studied extracts exhibited pronounced antioxidant activities, moderate anti-apoptotic and hepatoprotective potential. In silico docking experiments revealed that the compounds abundant in the extracts were able to bind to Bcl-2: Bim (BH3) interaction surface with an appreciable binding free energy.

**Discussion:**

The antioxidant and hepatoprotective activities exhibited by the studied extracts might be attributed to the high content of the polyphenols. The anti-apoptotic activity could be due to the interference with the apoptotic pathway mediated by Bcl-2: Bim interaction. These findings support the medicinal relevance of *Terminalia bellirica* and *Terminalia sericea* and provide a rational base for their utilization in folk medicine.

## Introduction

Plants have served as an essential source of drugs and remedies against diseases and health disorders since ancient times (Dias, Urban & Roessner, 2012). Polyphenol containing plants, which are common among medicinal plants, have reportedly shown various health benefits and applications. They have a wide spectrum of pharmacological activities including antioxidant, anti-inflammatory, anti-cancer, hepatoprotective, and antimicrobial activities. Polyphenols have been also proven to lower the risk of cardiovascular diseases, enhance liver regeneration, and increase life expectancy (van Wyk & Wink, 2015, 2017).

Liver damage can be life threatening. It may be caused by several factors such as viruses, alcohol, organic chemicals, metabolic, and genetic abnormalities (Robin et al., 2012). Liver transplantations have improved survival rate of patients but this treatment is limited to a small number of patients due to non-availability of suitable donors. Therefore, finding new remedies that are able to enhance liver regeneration and counteract liver failure is an imperative need. Natural products and plant extracts exhibiting antioxidant and hepatoprotective properties can be useful in this regards (van Wyk & Wink, 2015).

The genus *Terminalia* belongs to the family Combretaceae and consists of 200 tropical trees and shrubs, which are widely distributed in the tropical regions. Plants from this genus have been used traditionally to treat several health disorders such as diarrhea, skin rashes, cancer, inflammation, and different bacterial infections (Bessong et al., 2004; Okatch et al., 2012; Cock, 2015; van Wyk & Wink, 2015).

*Terminalia sericea* Burch ex DC. is a shrub or medium-sized deciduous tree growing in wide areas of Africa (Eloff, Katerere & McGaw, 2008; Mongalo et al., 2016). Root and stem bark extracts of *Terminalia sericea* have shown promising antibacterial and antidiabetic activities (Fyhrquist et al., 2002, Nkobole et al., 2011).

The phytochemical investigation of *Terminalia sericea* root extracts has revealed several compounds such as phenolic acids, saponins, lignans, triterpenoids, resveratrol glycosides, arjungenin, β-sitosterol, and stigmasterol (Bombardelli et al., 1974, Eldeen et al., 2006, Joseph et al., 2007).

*Terminalia bellirica* Roxb. is a deciduous tree that is widely distributed in the tropical regions. The fruit extract of *Terminalia bellirica* has shown hepatoprotective (Jadon, Bhadauria & Shukla, 2007) and anti-hypercholesterolemia activities (Shaila, Udupa & Udupa, 1995).

Several compounds such as lignans, ellagic, chebulagic, bellaric, and triterpene acids have been isolated from the fruit and stem bark extracts of *Terminalia bellirica* (Row & Murty, 1970, Nandy et al., 1989, Mahato, Nandy & Kundu, 1992, Valsaraj et al., 1997).

The aim of the current study was to identify the secondary metabolites in the leaf extracts of *Terminalia sericea* and *Terminalia bellirica* using HPLC-PDA-MS/MS. We also investigated the possible antioxidant, anti-apoptotic, and hepatoprotective activities of the extracts. Furthermore, we conducted a molecular modeling study to elucidate the mechanism of the anti-apoptotic activities of the extracts.

## Materials and Methods

### Plant material

Fresh mature plant leaves of *Terminalia bellirica* were collected from trees growing in Bangladesh Agricultural University Campus, Mymensingh, Bangladesh during the spring season. As for *Terminalia sericea*, the leaves were collected from the trees at Lupaga Site in Shinyanga, Tanzania. Voucher specimens of leaf samples are kept at IPMB, Heidelberg University under accession numbers P8654 and P7332 for *Terminalia bellirica* and *Terminalia sericea*, respectively.

The leaves of *Terminalia bellirica* (250 g) and *Terminalia sericea* (250 g) were air-dried, ground, and extracted with 100% methanol at room temperature for three days (6 × 500 mL). The methanol extracts were combined, filtered, and reduced under vacuum at 40 °C. After freezing at −70 °C, the extracts were freeze-dried (lyophilized) yielding fine dried powder with a yield of 10% and 12% for *Terminalia bellirica* and *Terminalia sericea*, respectively.

### HPLC-PDA-MS/MS

ThermoFinnigan LCQ-Duo ion trap mass spectrometer (ThermoElectron Corporation, Waltham, MA, USA) with an ESI source (ThermoQuest Corporation, Austin, TX, USA) was used to investigate the phytochemical composition of the leaves extracts of both *Terminalia* species. A C18 reversed-phase column (Zorbax Eclipse XDB-C18, Rapid resolution, 4.6 × 150 mm, 3.5 µm, Agilent, Santa Clara, CA, USA) was employed with a ThermoFinnigan HPLC system. The mobile phase was composed of water, acetonitrile (ACN, Sigma-Aldrich GmbH, Steinheim, Germany) and 0.1% formic acid. Initially ACN was 5% then increased to 30% over 60 min. The flow rate was kept at one mL/min with a 1:1 split before the ESI source. The extracts were injected using ThermoQuest surveyor autosampler. Xcalibur was utilized as operating software (Xcalibur™ 2.0.7; Thermo Fischer Scientific, Waltham, MA, USA). The MS operated in the negative mode as reported before (Sobeh et al., 2017a). The ions were detected in a full scan mode and over a mass range of 50–2,000 *m/z*. The compounds were identified based on their retention times, molecular weights, and fragmentation patterns as well as comparison with reported data from the same plant, available literature, and authentic compounds.

### Biological experiments

#### In vitro antioxidant activities

The Folin-Ciocalteu method, DPPH, and FRAP assays were carried out as previously described (Ghareeb et al. 2017).

### Evaluation of hepatoprotective effect

#### Animals

Adult male Wistar rats with body weight of 220 ± 30 g (10 weeks old) were obtained from the Faculty of Veterinary medicine, Zagazig University to be used in the current study. The rats were housed in polypropylene cages in an environmentally controlled breeding room with a temperature range of 18–22 °C, humidity range of 50–70%, and 12 h light/dark cycle. They were allowed free access to food and water. Animal procedures in the present study were performed according to the guidelines of the US National Institutes of Health on animal care and use and were approved by the ethical committee of the faculty of pharmacy, Zagazig University for animal use, approval number P6-6-2016.

### Induction of liver damage

Acute liver failure was induced by intraperitoneal injection of 800 mg/kg dose of D-galactosamine (D-GalN) dissolved in normal saline just before use (Mahmoud et al., 2014). After one week of acclimatization, 42 rats were randomly distributed into seven groups with six animals per group. Group 1 received vehicle (normal saline, 0.9% NaCl) for the whole duration of the experiment and served as the control group. Group 2 received vehicle for three days then, injected with 800 mg/kg D-GalN intraperitoneally. Groups 3 and 4 received *Terminalia sericea* in an oral dose of 100 and 200 mg/kg, respectively. Groups 5 and 6 received *Terminalia bellirica* in an oral dose of 100 and 200 mg/kg, respectively. Group 7 received the hepatoprotective standard drug, silymarin in an oral dose of 100 mg/kg and served as positive control. Groups 3–7 received the corresponding drugs or extracts once daily for three consecutive days before injecting D-GaIN (800 mg/kg) dissolved in normal saline.

### Blood and tissue sampling

Blood samples were obtained from the retro-orbital plexus 24 h after D-GalN injection. The blood was centrifuged (3,000 *g*, 4 °C, 20 min) to separate serum. The obtained serum was used to determine liver injury markers and total bilirubin. Thereafter, animals were injected with ketamine for anesthesia and euthanized by decapitation. The animals were dissected; livers were isolated and washed with cold saline to remove clotted blood. Each liver was dissected into two parts. The first part was placed in buffered formalin for further histopathological examination and the second part was flash frozen in liquid nitrogen and stored at −80 °C for determination of oxidative stress markers.

### Biochemical analysis

Markers of liver injury including serum aspartate aminotransferase (AST), alanine aminotransferase (ALT) activities, and total bilirubin level were measured colorimetrically (JENWAY 6105 UV/V Spectrophotometer) based on the method of Young (1995) and Balistreri & Shaw (1987), respectively and using a commercial assay kit (Diamond Diagnostic, Cairo, Egypt). Malondialdehyde (MDA) was measured according to the method of Ohkawa, Ohishi & Yagi (1979) using commercially available kits provided by Biodiagnostic (Giza, Egypt). Measurements of total antioxidant capacity (TAC) were performed as described by (Koracevic et al., 2001). In brief, TAC was measured after the reaction of the endogenous antioxidants in serum of rats with certain amount of hydrogen peroxide (H_2_O_2_). The residual H_2_O_2_ was measured by reaction with 3,5-dichloro-2-hydroxy benzene sulfonate yielding a colored product, which was detected by spectrophotometry (JENWAY 6105 UV/V Spectrophotometer) at a wavelength of 505 nm.

### Histopathological study

The isolated liver tissues were sliced and placed in 10% buffered formalin, dehydrated in ascending concentrations of ethyl alcohol (70%, 90%, and 100%), transferred to xylene, and embedded in paraffin wax. A rotary microtome was used to prepare liver sections of five μm thickness that were stained with H&E stain and examined for histopathological changes under a light microscope (Olympus BX-50 Olympus Corporation, Tokyo, Japan) (Bancroft, Stevens & Turner, 1996).

### Immunohistochemical study

The expression of the anti-apoptotic marker Bcl-2 was determined in liver tissues according to (Ozen et al., 2008). Briefly, the obtained liver tissues were deparaffinized in xylene and dehydrated using ethanol. The endogenous peroxidase activity was blocked by the addition of l.5% H_2_O_2_ in absolute methanol to the liver tissues which were then washed by phosphate buffered saline (PBS) before the addition of Bcl-2 primary antibodies. Streptavidin biotin peroxidase kit was used to measure protein expression. The Bcl-2 chromogen diaminobenzidine (DAB) was used to stain tissues and was then counterstained with hematoxylin for the protein detection (Zhou et al., 2005).

### Morphometric analysis

Five randomly high power microscopic fields were selected to count the number of hepatocytes positive to Bcl-2. They were analyzed using computerized image system (a Leica Qwin 500 image analyzer connected to a Leica microscope). This number represents cell number per µm² (Salama et al., 2013).

### Statistical analysis

All values of the hepatoprotective study are presented as mean ± standard error of mean. Statistical difference between various groups was determined using GraphPad Prism software (GraphPad, version 5; GraphPad Inc., La Jolla, CA, USA). Statistical significance of the results was performed using one-way analysis of variance test (ANOVA) followed by Tukey’s post hoc test to determine statistical difference between each two separate groups. A *p* value of <0.05 was considered as statistically significant.

## Results

### HPLC-PDA-MS/MS identification of secondary metabolites

**Figure 1:**
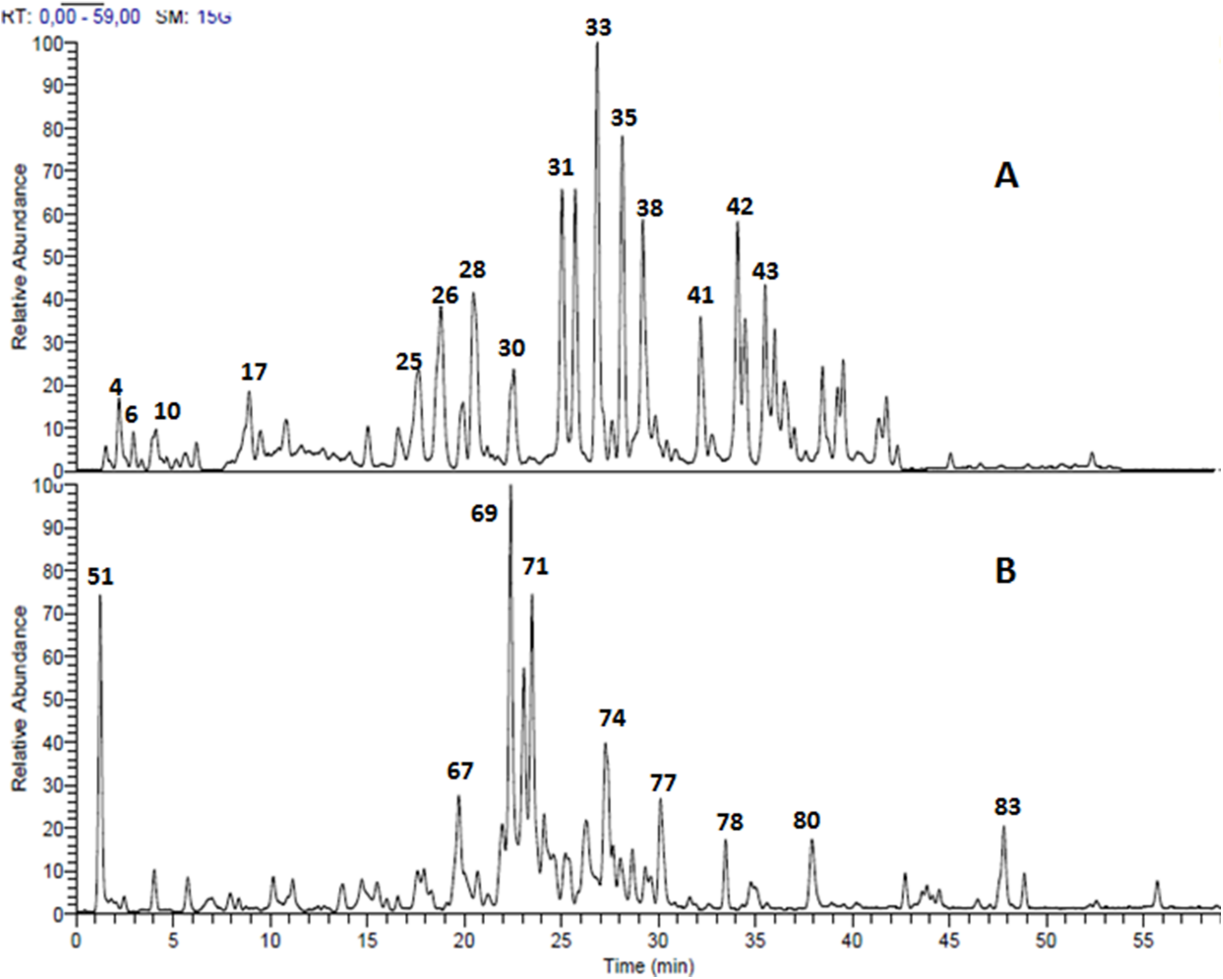
LC-MS chromatogram. Total ion chromatogram of the methanol leaves extracts (A) *Terminalia bellirica*, 50 compounds were separated; (B) *T. sericea*, 35 compounds were separated.

Structural analysis of polyphenolic compounds found in the leaf extracts of *Terminalia bellirica* and *Terminalia sericea* resulted in the separation and tentative identification of 85 compounds in total. Ellagitannins and proanthocyanidins represented the major chemical composition of *Terminalia bellirica* leaves extract while flavonoid glycosides and stilbenoids were the most abundant phytochemicals in *Terminalia sericea* leaves extract. The identified compounds along with their retention times, observed molecular and fragment ions are listed in Table 1. LC-MS profiles for *Terminalia bellirica* and *Terminalia sericea* leaves extracts in the negative ion mode are shown in Fig. 1.

**Table 1:** Chemical composition of the methanol extracts of *Terminalia bellirica* and *T. sericea* leaves.

No.	Rt(min)	[M−H]^−^*m/z*	MS/MS	Tentatively identified compound	Relative abundance	References
***Terminalia bellirica***						
1	1.52	191	127	Quinic acid	0.21	(Sobeh et al., 2017a)
2	1.72	133	115	Malic acid	0.22	
3	1.77	173	155, 111	Shikimic acid	tr.	
4	2.35	481	301	Hexahydroxydiphenoyl (HHDP) hexoside^a^	1.14	(Khan et al., 2017)
5	2.88	331	169, 193	Galloyl-hexoside^a^	0.79	(Khan et al., 2017)
6	2.93	481	301, 257	HHDP hexoside	0.73	(Khan et al., 2017)
7	3.37	331	169	Galloyl-hexoside^a^	tr.	(Khan et al., 2017)
8	3.84	783	765, 685, 301	bis-HHDP-hexoside	tr.	(Zhu, Dong & Guo, 2015)
9	3.89	169	125	Gallic acid*	tr.	(Fahmy et al., 2016)
10	4.09	633	301, 257, 249	Punicacortein	1.56	
11	4.25	609	441, 305	(epi)Gallocatechin-(epi)gallocatechin	tr.	
12	4.66	483	331, 169	Digalloyl-hexoside	0.22	
13	6.23	633	301	Galloyl-HHDP-hexoside^a^	0.58	(Zhu, Dong & Guo, 2015)
14	8.23	593	441, 425, 289	(epi)Catechin-(epi)gallocatechin	0.24	
15	8.38	783	483, 301	bis-HHDP-hexoside	0.53	(Zhu, Dong & Guo, 2015)
16	8.64	305	287, 179	(epi)Gallocatechin	tr.	(Sobeh et al., 2017b)
17	8.94	801	757, 633, 399, 299	Punigluconin	1.44	
18	9.5	933	915, 781, 601, 299	Castalagin/Vescalagin	0.77	
19	10.79	593	441, 305	(epi)Catechin-(epi)gallocatechin	1.27	(Sobeh et al., 2017c)
20	12.67	593	441, 305	(epi)Catechin-(epi)gallocatechin	0.39	(Sobeh et al., 2017c)
21	14.01	633	301	Galloyl-HHDP-hexoside	0.40	(Zhu, Dong & Guo, 2015)
22	14.37	783	481, 301	bis-HHDP-hexoside	0.25	(Zhu, Dong & Guo, 2015)
23	15.01	1083	781, 601, 451, 423	Punicalagin	0.70	
24	16.54	183	169, 125	Methylgallate	1.17	(Marzouk et al., 2002)
25	17.56	951	907, 783, 481	Granatin B	4.03	
26	18.72	577	451, 425, 289	(epi)Catechin-(epi)catechin	6.32	(Sobeh et al., 2017a)
27	19.91	289	245, 205, 179	Catechin*	2.02	(Sobeh et al., 2017c)
28	20.52	785	633, 615, 483, 301	Digalloyl-HHDP-hexoside	6.40	(Wyrepkowski et al., 2014)
29	21.45	483	331, 271, 169	Digalloyl-hexoside	0.82	
30	22.53	291	247	Brevifolin carboxylic acid	3.24	(Fahmy et al., 2016)
31	25.10	785	633, 615, 483, 301	Digalloyl-HHDP-hexoside	6.69	(Wyrepkowski et al., 2014)
32	25.67	953	909, 785, 301	Chebulagic acid	6.47	(Fahmy et al., 2016)
33	26.77	633	463, 301, 229	Corilagin*	10.35	(Kinoshita et al., 2007)
34	27.19	469	425	Valoneic acid dilactone	0.80	(Wyrepkowski et al., 2014)
35	28.08	1235	1083, 781, 601	Galloylpunicalagin	7.44	(Chen et al., 2009)
36	28.30	729	577, 407, 289	Procyanidin dimer mono gallate	tr.	(Sobeh et al., 2017c)
37	28.56	865	739, 695, 577, 287	(epi)Catechin-(epi)catechin-(epi)catechin	tr.	(Sobeh et al., 2017c)
38	29.12	635	483, 465	Tri-galloyl-hexoside	7.41	(Ben Mansour et al., 2017)
39	29.96	967	785, 765, 483, 301	Ellagitannin	1.13	
40	30.43	577	559, 451, 425, 289	(epi)Catechin-(epi)catechin	0.33	(Sobeh et al., 2017b)
41	32.13	447	429, 357, 327, 285	Orientin*	3.76	(Fahmy et al., 2016)
42	33.98	937	785, 767, 465, 301	Punicafolin	5.78	
43	35.5	301	301, 257, 229	Ellagic acid*	tr.	(Fahmy et al., 2016)
44	36.03	431	269, 179, 161	Vitexin*	3.06	(Fahmy et al., 2016)
45	36.49	609	301, 271, 179	Quercetin coumaroyl-glucoside^a^	2.00	(Sobeh et al., 2017a)
46	37.03	599	447, 285	kaempferol galloyl-hexoside	0.40	(Sobeh et al., 2017a)
47	38.6	935	917, 765, 451, 301	Galloyl-bis-HHDP-hexoside	2.62	(Zhu, Dong & Guo, 2015)
48	39.45	953	937, 911, 785, 617	Chebulagic acid isomer	3.77	
49	41.84	447	315	Isorhamnetin pentoside	1.62	
50	42.33	483	301	1,6-Digalloyl glucose	0.48	
***T. sericea***						
51	1.22	355	337, 249	Chebulic acid	8.27	(Fahmy et al., 2016)
52	2.08	481	301	Hexahydroxydiphenoyl (HHDP) hexoside^a^	2.27	(Khan et al., 2017)
53	2.51	331	169	Galloyl-hexoside^a^	0.32	(Ghareeb et al., 2017)
54	3.72	483	331, 169	Digalloyl-hexoside	tr.	
55	3.98	291	247	Brevifolin carboxylic acid	1.09	(Fahmy et al., 2016)
56	8.02	483	313, 271, 169	Digalloyl-hexoside	tr.	
57	10.13	183	183, 169, 125	Methylgallate	1.43	(Marzouk et al., 2002)
58	10.19	289	245, 205, 179	(epi)Catechin*	0.55	(Sobeh et al., 2017b)
59	11.01	935	917, 783, 633, 301	Casuarictin	1.47	
60	12.48	325	265, 235, 163	5-*O*-Galloylshikimic acid	0.43	
61	13.69	467	313, 169	Gallic acid rhamnosyl-gallate	0.97	
62	14.68	633	463, 301, 257	Galloyl-HHDP-hexoside^a^	1.60	(Zhu, Dong & Guo, 2015)
63	14.78	955	937, 785, 633, 465	Chebulinic acid	1.06	(Fahmy et al., 2016)
64	15.49	633	463, 301, 257	Punicacortein A	0.90	
65	16.56	521	359	Rosmarinic acid glucoside	0.36	(Sobeh et al., 2017a)
66	17.67	635	483, 465, 313	Tri-galloyl-hexoside	1.86	(Fahmy et al., 2016)
67	19.60	625	317, 271, 179	Myricetin rutinoside	5.07	
68	19.79	479	317, 271, 179	Myricetin glucoside	0.88	(Sobeh et al., 2017a)
69	21.88	609	301, 271,179	Quercetin rutinoside	3.33	(Marzouk et al., 2002)
70	22.43	615	463, 301, 271	Quercetin galloyl-glucoside	24.25	(Sobeh et al., 2017a)
71	23.58	615	463, 301, 271	Quercetin galloyl-glucoside	8.28	(Sobeh et al., 2017a)
72	25.31	463	301, 179, 151	Quercetin glucoside	2.47	(Sobeh et al., 2017a)
73	26.21	433	301, 179, 151	Quercetin pentoside	5.47	(Sobeh et al., 2017a)
74	27.30	433	301, 179, 151	Quercetin pentoside	7.86	(Sobeh et al., 2017a)
75	28.61	433	301, 179, 151	Quercetin pentoside	1.44	(Sobeh et al., 2017a)
76	29.09	447	301, 179, 151	Quercetin rhamnoside	1.78	(Sobeh et al., 2017a)
77	30.12	447	301, 179, 151	Quercetin rhamnoside	3.84	(Sobeh et al., 2017a)
78	33.41	389	227, 185, 141	Resveratrol glucoside	1.75	
79	35.70	761	609, 301	Quercetin galloyl-rutinoside	1.72	
80	37.89	541	379, 227, 169	Resveratrol galloyl-glucoside	2.76	
81	42.72	609	463, 301, 179	Quercetin coumaroyl-glucoside^a^	0.90	(Sobeh et al., 2017a)
82	44.52	301	301, 179, 151	Quercetin	0.56	(Sobeh et al., 2017a)
83	47.83	535	389, 307, 227	Resveratrol coumaroyl-glucoside	3.15	
84	48.84	343	329	Methyltricin	0.88	
85	55.74	535	389, 307, 227	Resveratrol coumaroyl-glucoside	0.87	

**Notes:**

* Identification was further confirmed with available authentic standards.

a Compounds identified in both species.

tr.: Indicates that the relative abundance of the compound is less than 0.20%.

**Figure 2:**
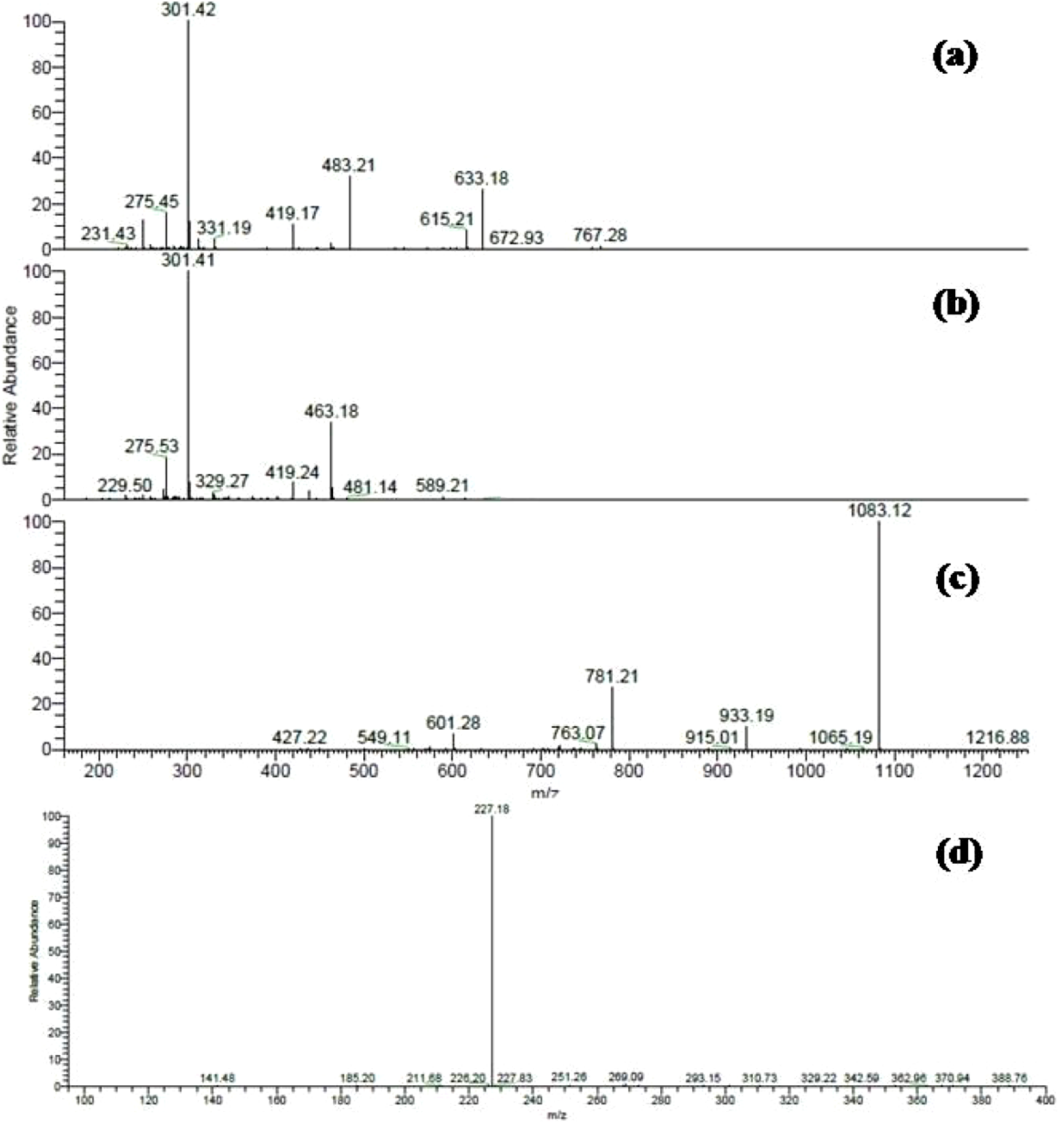
ESI-MS/MS spectra of some selected compounds in the extracts. ESI-MS/MS of (A) Digalloyl-HHDP-hexoside [M – H]^−^ at m/z 785; (B) Corilagin [M – H]^−^ at m/z 633; (C) Galloylpunicalagin [M – H]^−^ at m/z 1235; (D) Resveratrol glucoside [M – H]^−^ at m/z 389.

Several ellagitannins were detected in *Terminalia bellirica*. For instance, the deprotonated molecular ion peak of compound 31 showed [M – H]^−^ at *m/z* 785. The MS^2^ experiments revealed an ion at *m/z* 633 [M – H – 152], which was attributed to the loss of a galloyl group. Another ion was detected at *m/z* 483 indicating the removal of the hexahydroxydiphenoyl (HHDP) moiety. The ion at *m/z* 301 was formed due to the elimination of digalloylglucose [M – H – 483]. This fragmentation pattern suggested that the molecule is digalloyl-HHDP-hexoside (Fig. 2A) (Wyrepkowski et al., 2014).

Compound 33 was identified as corilagin. It displayed an [M – H]^−^ at *m/z* 633 with fragment ions at *m/z* 463 [M – H – 170] and *m/z* 301 [M – H – 170 – 162] (Fig. 2B) (Kinoshita et al., 2007). The identification was also confirmed using the available standard compound.

Compound 35 was identified as galloylpunicalagin (Fig. 2C) (Chen et al., 2009). It showed a molecular ion peak [M – H]^−^ at *m/z* 1235 and its MS^2^ spectrum revealed a main fragment ion at *m/z* 1083 which corresponded to punicalagin. This was confirmed by two fragments ions at *m/z* 781 and 601 as previously described by (Fahmy et al., 2016). The ESI-MS analysis of compound 38 displayed a [M – H]^−^ at *m/z* 635. It showed product ions at *m/z* 483, 465, and 313 in MS^2^ experiments and thus was identified as tri-galloyl-hexoside (Ben Mansour et al., 2017).

MS data analysis of *Terminalia sericea* leaves extract revealed the presence of myricetin, quercetin, and resveratrol derivatives as major constituents. Peaks numbered 70 and 71 were identified as quercetin galloyl-glucosides. They showed [M – H]^−^ at *m/z* 615 and fragment ions at *m/z* 463 [M – H – 152] and 301 [M – H – 152 – 162] as reported before (Sobeh et al. 2017a).

Several resveratrol derivatives were also detected. In particular, resveratrol glucoside was identified based on its [M – H]^−^ at *m/z* 389 and a daughter ion at *m/z* 227 [M – H – 162], (Fig. 2D). Other two peaks showed a deprotonated ion at *m/z* 535 and two daughter ions at *m/z* 389 and 227 were characterized as resveratrol coumaroyl-glucoside (Table 1).

### Antioxidant activity and total phenolic content

*Terminalia bellirica* and *Terminalia sericea* leaves extracts exhibited substantial antioxidant activity in the DPPH and FRAP assays when compared to the well-known antioxidant epigallocatechin gallate (EGCG) from green tea. Folin-Ciocalteu assay revealed high total phenolic content in the studied extracts (Table 2).

**Table 2:** DPPH and FRAP activities and total phenolic content of the methanol extracts from *T. bellirica* and *T. sericea* leaves.

Leaf extract	DPPH	FRAP	Total phenolic content
	IC_50_, µg/mL	mM FeSO_4_/mg extract	µg gallic acid equivalent/mg extract
*T. bellirica*	2.6 ± 19	19.26 ± 0.14	458
*T. sericea*	5.60 ± 0.57	18.20 ± 0.10	418
EGCG	2.85 ± 0.16	25 ± 0.21	–

**Note:**

EGCG: Epigallocatechin gallate as a positive control.

### Hepatoprotective potential

**Figure 3:**
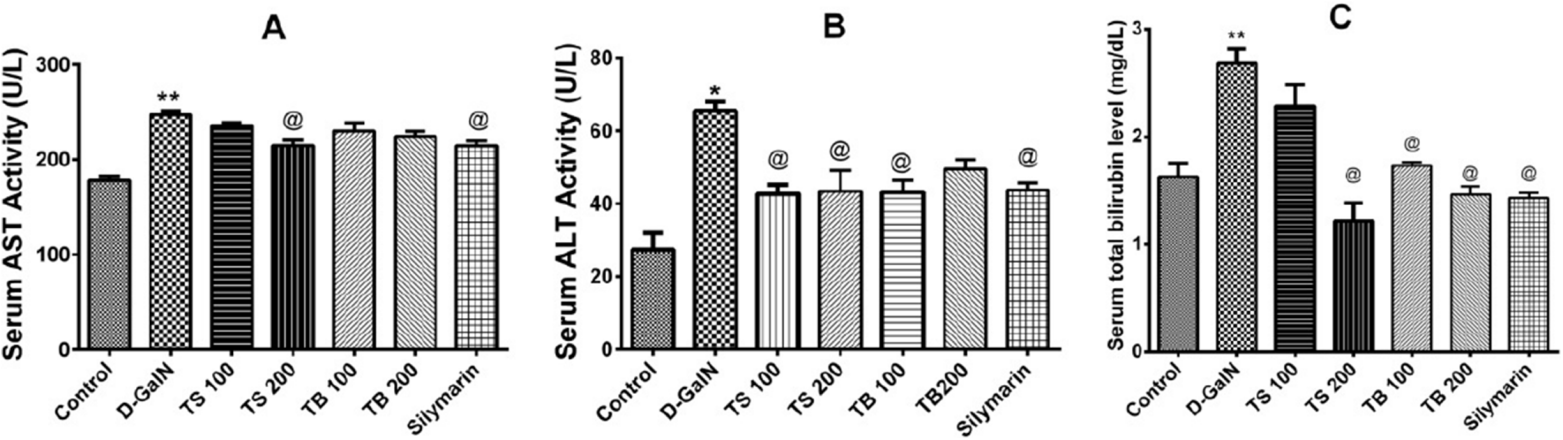
Hepatoprotective activities. Effects of *T. sericea* extract (TS, 100 mg/kg and 200 mg/kg, oral), *T. bellirica* (TB, 100 mg/kg and 200 mg/kg, oral), and silymarin on D-GalN (800 mg/kg, i.p.) induced hepatotoxicity. (A) AST activities (aspartate aminotransferase); (B) ALT activities (alanine aminotransferase); (C) Total bilirubin level. Each value represents mean ± SEM of six animals. *Significantly different from untreated control group at *p* < 0.001, **Significantly different from untreated control group at *p* < 0.0001, ^@^Significantly different from D-GalN group at *p* < 0.05. One-way ANOVA followed by Tukey post hoc test was used as statistical test.

The effects of *Terminalia sericea* and *Terminalia bellirica* leaves extracts (100 and 200 mg/kg dose levels) and the positive control silymarin from *Silybum marianum* (100 mg/kg) on D-GalN-induced hepatic toxicity in rats were evaluated. A significant increase in the serum levels of AST (*p* < 0.0001), ALT (*p* < 0.001) and total bilirubin (*p* < 0.0001) was observed in the D-GalN treated rats compared to the control group (Fig. 3). Pre-treatment with *Terminalia sericea* (100 and 200 mg/kg b.w.), *Terminalia bellirica* (100 mg/kg), and silymarin (100 mg/kg) caused significant reduction in the levels of ALT compared to D-GalN group (*p* < 0.05). There was no significant difference between the extracts and silymarin (*p* > 0.05). On the other hand, only *Terminalia sericea* (in a dose of 200 mg/kg) was able to decrease serum AST level compared to D-GalN group (*p* < 0.05). The low dose level of *Terminalia sericea*, the two dose levels of *Terminalia bellirica* and silymarin did not significantly affect AST enzyme activity compared to D-GalN group (*p* > 0.05). *Terminalia sericea* in the high dose level, *Terminalia bellirica* in the two dose levels and silymarin decreased total bilirubin level when compared to D-GalN group.

**Figure 4:**
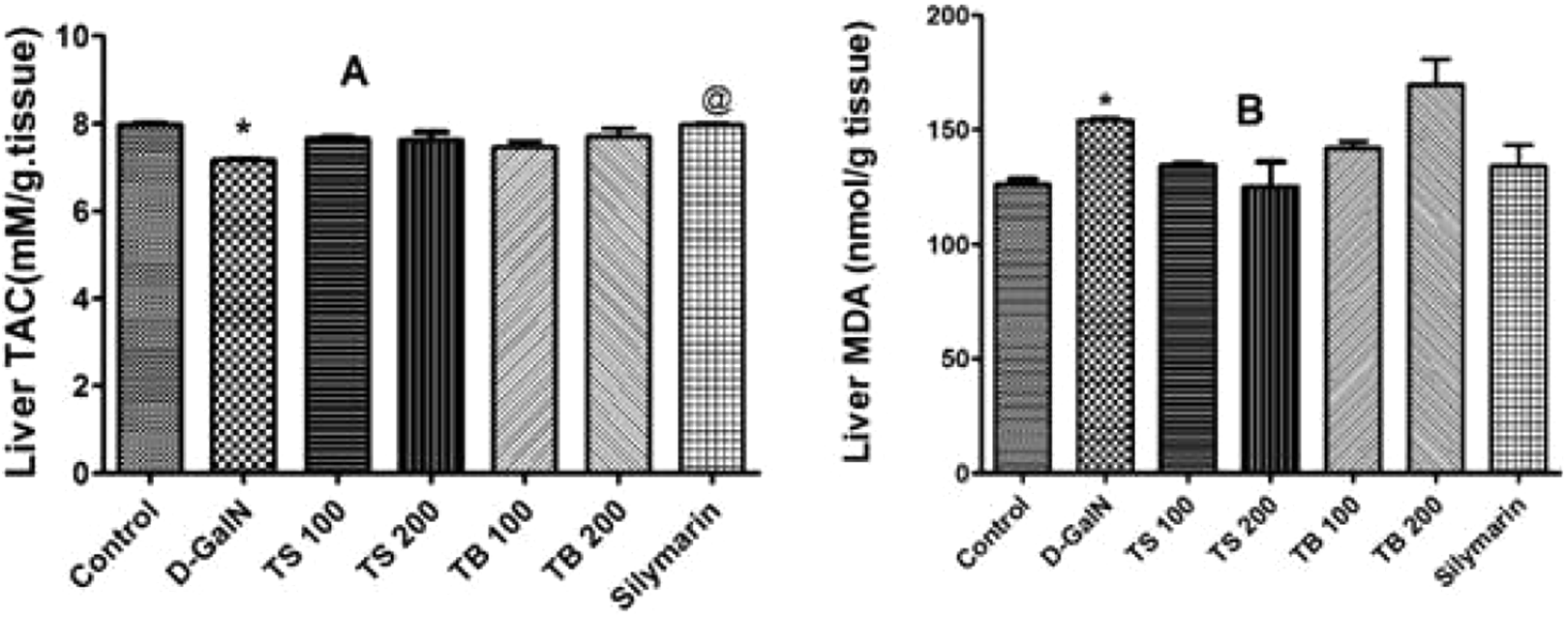
Antioxidant activities in animals. Effects of *T. sericea* extract (TS, 100 mg/kg and 200 mg/kg, oral), *T. bellirica* (TB, 100 mg/kg and 200 mg/kg, oral), and silymarin on D-GalN (800 mg/kg, i.p.) induced oxidative stress. (A) TAC (total antioxidant capacity, mM/g tissue); (B) MDA (malondialdehyde, nmol/g tissue) in the liver. Each value represents mean ± SEM of six rats. *Significantly different from control group at *p* < 0.001, ^@^Significantly different from D-GalN treatment group at *p* < 0.0007. *n* = 6; One-way ANOVA followed by Tukey post hoc test was used as a statistical test of significance.

D-galactosamine intoxication led to a decrease in the total antioxidant capacity as shown in Fig. 4A. Both extracts failed to restore the total antioxidant capacity (*p* > 0.05). Silymarin on the other hand was able to show an improvement when compared to D-GalN group (*p* < 0.0007).

Lipid peroxidation in hepatic tissues, manifested as elevated production of MDA, was increased significantly relative to the control group (*p* < 0.0018) upon administration of D-GalN (Fig. 4B). No change in the level of MDA was observed in all pre-treated groups relative to D-GalN group.

### Structural changes of the liver

**Figure 5:**
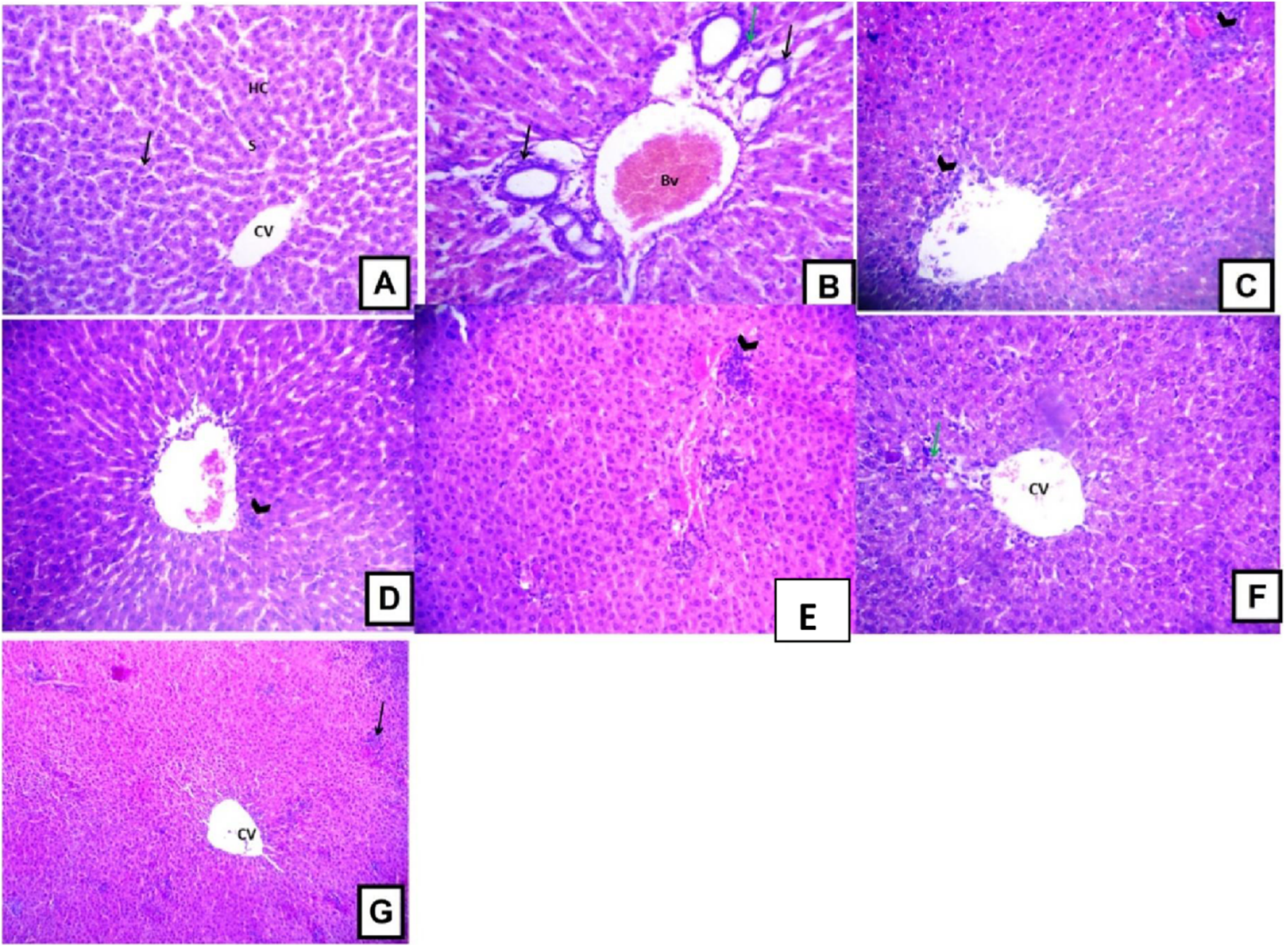
Hepatoprotective activities. Effects of *T. sericea* extract (TS, 100 mg/kg and 200 mg/kg, oral), *T. bellirica* (TB, 100 mg/kg and 200 mg/kg, oral), and silymarin on D-GalN-induced histopathological changes in rats (H&E staining; 200x). (A) a liver section of a rat from the control group, showing normal liver tissues, normal hepatic cords (HC) radiating from the central vein (CV) and normal blood sinusoids (S) and normal hepatocytes (arrow); (B) a liver section of one rat of D-GalN group, mononuclear cellular infiltration in the portal areas (arrow head) and congestion of blood vessels (BV), and hyperplasia of bile ducts (black arrow); (C) represents *T. sericea* extract (100 mg/kg) pre-treated rat followed by D-GalN showing mononuclear cellular infiltration (arrow heads) and small area of hemorrhage (Hg); (D) Liver section of an adult rat pre-treated with *T. sericea* extract (200 mg/kg) followed by D-GalN group showing marked improvement with little mononuclear cellular infiltration (arrow head).There are multiple areas with mononuclear cellular infiltration (arrows) and congested central vein (CV); (E) Liver pre-treated with *T. bellirica* (100 mg/kg) followed by D-GalN group showing the portal tract area in which there is mononuclear cellular infiltration (arrow head) and congested blood vessel (Bv); (F) Liver pre-treated with *T. bellirica* (200 mg/kg) followed by D-GalN group showing marked improvement with little mononuclear cellular infiltration (arrow head); (G) represents silymarin (100 mg/kg) pre-treated rat followed by D-GalN showing some areas with mononuclear cellular infiltration (arrow) and partial improvement.

Histopathological examination of the liver sections from the control group revealed the usual liver architecture. The hepatic lobules and central veins were normal (Fig. 5A). Liver tissues isolated from D-GalN treated group showed mononuclear cell infiltration in the portal area with congested blood vessels and hyperplasia of bile ducts (Fig. 5B). Liver sections from *Terminalia sericea* (100 mg/kg) pre-treated rats showed mononuclear cellular infiltration and small areas of hemorrhage (Fig. 5C). Liver sections from *Terminalia bellirica* (100 mg/kg) pre-treated rats showed mononuclear cellular infiltration in the portal area and congested blood vessels (Fig. 5E). Meanwhile, liver sections from rats pre-treated with *Terminalia sericea* and *Terminalia bellirica* in the high dose level (200 mg/kg) showed marked improvement with little mononuclear cellular infiltration (Figs. 5D and 5F). Silymarin pre-treated rats showed partial improvement of the structural changes (Fig. 5G).

### Effect on Bcl-2 expression

**Figure 6:**
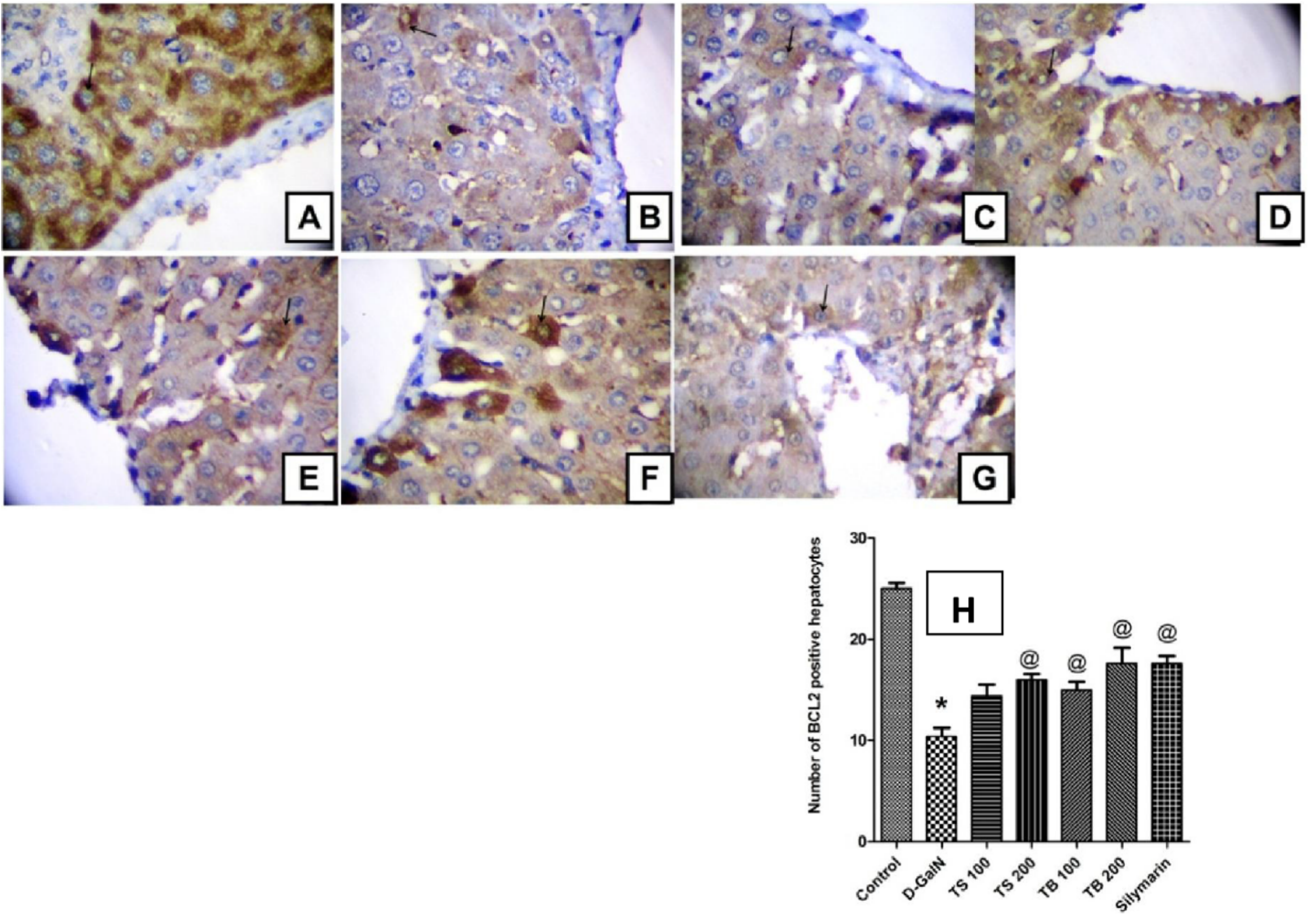
Effect on Bcl-2 expression. Immunohistochemical determination of Bcl-2 expression in paraffin-embedded liver tissues stained with Avidin-biotin peroxidase stain with hematoxylin counter stain, [*n* = 5; 400× original magnification]. (A) control group withstrong positive cytoplasmic Bcl-2 immunoreaction (arrow); (B) D-GalN group, with weak cytoplasmic immunoreaction for Bcl-2 (arrow); (C) *T. sericea* extract (100 mg/kg) pre-treated rat followed by D-GalN showing mild cytoplasmic immunoreaction for Bcl-2 (arrow); (D) Liver section of rat pre-treated with *T. sericea* extract (200 mg/kg) followed by D-GalN group showing moderate cytoplasmic immunoreaction for Bcl-2 (arrow); (E) Liver section of an adult rat pre-treated with *T. bellirica* (100 mg/kg) followed by D-GalN showing mild cytoplasmic immunoreaction for Bcl-2 (arrow) showing mild cytoplasmic immunoreaction for Bcl-2 (arrow); (F) A liver section of an adult rat pre-treated with *T. bellirica* (200 mg/kg) followed by D-GalN showing moderate cytoplasmic immunoreaction for Bcl-2 (arrow); (G) A liver section of an adult rat pre-treated with silymarin (100 mg/kg) followed by D-GalN showing moderate cytoplasmic immunoreaction for Bcl-2 (arrow); (H) The column graph reveals morphometric analysis of the mean number of hepatocytes, which are positive to Bcl-2 ± SEM, (*n* = 5), One-way ANOVA followed by Tukey *post hoc* test was used to detect statistical difference. *Significantly different from control group at *p* < 0.05%, ^@^Significantly different from D-GalN group at *p* < 0.0001%.

The anti-apoptotic Bcl-2 protein promotes survival and prevents apoptotic cell death. The current study revealed that D-GalN administration was able to induce free radical generation and decrease the endogenous antioxidants. This was in turn associated with reduction in the number of hepatocytes with positive Bcl-2 expression indicating increased apoptotic cell death (Fig. 6). All extract doses, except the low dose level of *Terminalia sericea*, were able to increase the positive Bcl-2 expression hepatocytes indicating protection against apoptosis. This effect did not significantly differ from that of silymarin on Bcl-2 expression (*p* < 0.0001).

### Molecular modeling

To verify their ability to interfere with apoptosis and minimize hepatic cell death, the most abundant compounds identified in the investigated extracts were docked into Bcl-2: Bim (BH3) interaction surface, pdb code: 4b4s, with the help of molecular operating environment, 2013.08; Chemical Computing Group Inc., Montreal, QC, Canada, H3A 2R7, 2016 according to our previously applied protocol (Sobeh et al., 2017d). The selected compounds and their docking results are shown in Table 3.

**Table 3:** Scoring function and amino acids interactions of the docked compounds into Bcl-2: Bim (BH3) interface.

Number	Compound name	Scoring function	Amino acids interactions
25	Granatin B	−14.90	Tyr 73 (H-bonding) Phe 159 (H-bonding) Arg 39 (H-bonding through solvent)
30	Brevifolin carboxylic acid	−14.18	Arg 44 (ionic) Arg 44(H-bonding) Tyr 73 (H-bonding) Arg44 (Hydrophobic)
32	Chebulagic acid	−17.70	Tyr 73 (H-bonding) Arg160 (H-bonding)
33	Corilagin	−15.60	Tyr 73 (H-bonding)
38	Tri-galloyl-hexoside	−15.62	Tyr 72 (Hydrophobic) Arg 44 (Arene-Cation)
41	Orientin	−16.26	Tyr73 (H-bonding) Arg 44 (H-bonding) Phe 159 (H-bonding through solvent) Thr 161 (H-bonding through solvent)
42	Punicafolin	−24.44	Tyr 73 (H-bonding) Arg 160 (H-bonding) Ala36 (H-bonding through solvent)
43	Ellagic acid	−12.38	Ser 40 (Hydrophobic) Arg 160 (Hydrophobic)
67	Myricetin rutinoside	−17.34	Gln 47 (H-bonding) Tyr 73 (H-bonding) Phe 159 (H-bonding through solvent) Arg 160 (H-bonding through solvent) Thr161 (H-bonding through solvent) Phe 163 (H-bonding through solvent)
68	Myricetin glucoside	−11.32	Tyr 73 (H-bonding)
69	Quercetin rutinoside	−16.45	Tyr 73 (H-bonding) Phe 159 (H-bonding through solvent) Ser 40 (H-bonding) Arg 44 (H-bonding)
77	Quercetin rhamnoside	−15.11	Ser 40 (H-bonding) Arg 44 (Arene-Cation) Tyr 73 (H-bonding) Phe 163 (Hydrophobic)
78	Resveratrol glucoside	−13.98	Ile 48 (Hydrophobic) Tyr 73 (H-bonding)
80	Resveratrol galloyl glucoside	−12.04	Ser 40 (H-bonding) Tyr 73 (H-bonding) Arg 160 (Hydrophobic)

It was reported that a heterodimerization of pro- and anti-apoptotic two protein members into a complex is crucial to activate the apoptotic pathways (Reed, 2000). The surface of the anti-apoptotic Bcl-2 has a hydrophobic cleft to which the BH3 domain of apro-apoptotic protein such as Bim binds. The BH3 domain of Bim extends from Arg 53 to Ala 74 residues. Out of these amino acids, five hydrophobic residues, namely, Ile 58, Leu 62, Ile 65, Phe 69, and Tyr 73 were reported to interact with the Bcl-2 hydrophobic cleft and are conserved in all the pro-apoptotic BH3 domains (Rautureau et al., 2012). Other residues such as Arg 64, Glu 68, and Tyr 72 were reported to contribute with different degrees to the complex binding free energy (Delgado-Soler et al., 2012). Introduced mutation to any of these residues was reported to block apoptosis as it impairs severely the binding of the two proteins (Sattler et al., 1997; Boersma et al., 2008).

**Figure 7:**
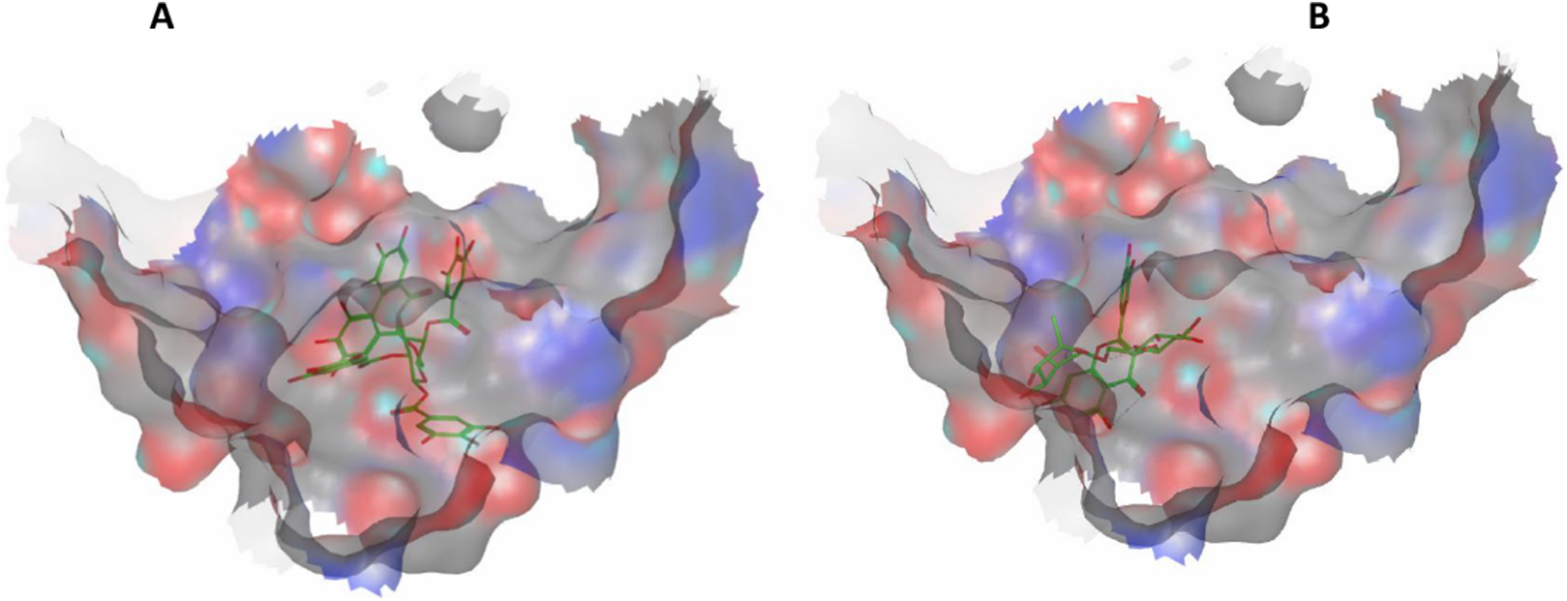
Molecular modeling. 3D docking poses of compounds 42 (A) and 69 (B) docked into Bcl-2: Bim (BH3) interface.

**Figure 8:**
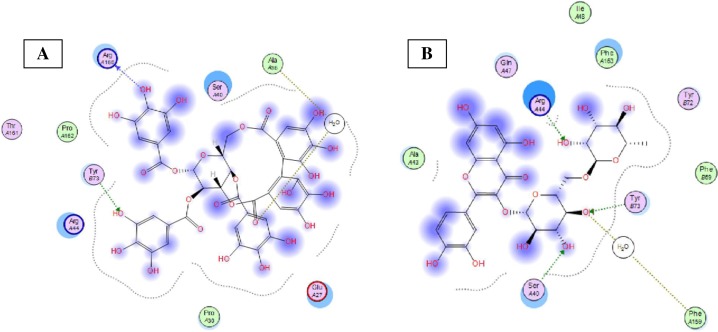
Molecular modeling. 2D interactions of compounds 42 (A) and 69 (B) with amino acid residues in Bcl-2: Bim(BH3) interface.

Eight highly abundant compounds in *Terminalia sericea* and *Terminalia bellirica* leaves extracts were selected for docking into Bcl-2: Bim (BH3) interaction surface. As shown in Table 3, all compounds were able to bind to the binding site with an appreciable scoring function range of – 11.32 to – 24.44. The docked compounds have shown different polar and hydrophobic interactions with the amino acids in the binding site, most importantly Tyr 73 and/or Tyr 72. These interactions could result in changing the protein structural conformation leading to interference with the binding of Bim (BH3) to Bcl-2 and thus blocking apoptosis. Among the glycosides of *Terminalia sericea*, myricetin rutinoside and quercetin rutinoside have shown the best score of –17.34 and –16.45 respectively followed by the more rigid—by virtue of the alkene moiety in its side chain—resveratrol glucoside, which showed a scoring function of –13.98. As for *Terminalia bellirica*, the bulky compounds containing hexahydroxydiphenoyl and/or dehydrohexahydroxydiphenoyl units such as punicafolin, corilagin, granatin B, and the benzopyran tannin chebulagic acid have shown the best scores of –24.44, –15.6, –14.9, and –17.7 respectively while the small sized ellagic acid has shown the weakest binding among this group with –12.38 scoring function. Figures 7 and 8 show the 3D poses and 2D-interactions of compounds 42 and 69 as selected examples of the docked compounds.

## Discussion

In general, both *Terminalia bellirica* and *Terminalia sericea* demonstrated moderate hepatoprotective effects. *Terminalia sericea* exerted effects that were comparable to those of silymarin on liver enzymes and bilirubin. Similar results had been reported from other polyphenol-rich extracts. A study on *Turraea fischeri* bark extract reported pronounced hepatoprotective effect that was attributed to its polyphenol content (Sobeh et al., 2017c).

In another study, a *Cassia abbreviata* extract that is rich in proanthocyanidins has exhibited potent antioxidant and hepatoprotective activities (Sobeh et al., 2018a). Furthermore, a previous study performed on *Ximenia americana* var. *caffra* rich in tannins has shown potent antioxidant, antidiabetic, and hepatoprotective effects (Sobeh et al. 2017e). These previous findings are in agreement with the results in the current study.

Both *Terminalia* species exhibited an anti-apoptotic effect by elevating the expression of the anti-apoptotic protein Bcl-2. This might be attributed to ellagitannins and proanthocyanidins. Similar results were reported from a study on *Senna sengueana* bark extract, rich in proanthocyanidins (Sobeh et al., 2017b). Different crude extracts having high polyphenol contents such as *Terminalia bellirica*, *Terminalia chebula*, *Terminalia myriocarpa*, and other *Terminalia* species have shown comparable antioxidant and hepatoprotective properties (Marzouk et al., 2002; Kinoshita et al., 2007; Pfundstein et al., 2010; Fahmy et al., 2016).

## Conclusions

The polyphenol composition in *Terminalia sericea* and *Terminalia bellirica* was characterized utilizing HPLC-PDA-MS/MS. 50 compounds were tentatively identified in *Terminalia bellirica* extract, mainly ellagitannins and proanthocyanidins, whereas 35 secondary metabolites were annotated from the leaves extract of *Terminalia sericea*, mainly flavonoid glycosides and stilbenoids. Both extracts exhibited robust antioxidant properties, moderate hepatoprotective, and anti-apoptotic activities. In addition, the abundant compounds in both extracts were able to bind to Bcl-2: Bim (BH3) interaction surface and showed different polar and hydrophobic interactions. They could result in changing the protein structural conformation leading to interference with the binding of Bim (BH3) to Bcl-2 and thus blocking apoptosis. These findings need to be more explored in more detail and investigated through further set of experiments.

## Supplemental Information

10.7717/peerj.6322/supp-1 Supplemental Information 1 Raw data: Antioxidant activities from both extracts.

10.7717/peerj.6322/supp-2 Supplemental Information 2 Hepatoprotective raw data from both extracts.

## Abbreviations

ANOVA: Analysis of variance
ALT: Alanine aminotransferase
AST: Aspartate aminotransferase
D-GaIN: D-galactosamine
DPPH: 2,2-diphenyl-1-picrylhydrazyl
FRAP: Ferric reducing antioxidant power
GGT: gamma-glutamyltransferase
GSH: Reduced glutathione content
HPLC-PDA-ESI-MS/MS: High-performance liquid chromatography-photodiode array detector-electrospray ionization mass spectrometry
MDA: Malondialdehyde

## Additional Information and Declarations

### Data Availability

The following information was supplied regarding data availability: Raw data are available in the Supplemental Files.
